# Thyrotoxic Periodic Paralysis: A Rare Cause of Quadriparesis in a Young and Seemingly Healthy Patient

**DOI:** 10.3390/medicina60101685

**Published:** 2024-10-14

**Authors:** Adrian-Gabriel Florescu, Evelina-Ioana Galeș, Sabina Adriana Frunză, Camelia Cristina Diaconu

**Affiliations:** 1 Department of Internal Medicine, Clinical Emergency Hospital of Bucharest, 105402 Bucharest, Romania; evelinastrugaru@yahoo.com (E.-I.G.);; 2 Faculty of Medicine, University of Medicine and Pharmacy Carol Davila Bucharest, 050474 Bucharest, Romania; 3 Academy of Romanian Scientists, 050045 Bucharest, Romania

**Keywords:** hypokalemia, thyrotoxic periodic paralysis, Graves’ disease, thyrotoxicosis

## Abstract

Hypokalemia is a common laboratory finding in hospitalized patients, typically resulting from insufficient potassium intake, renal or gastrointestinal losses, or intracellular shifts. While the underlying cause is often easily identifiable, certain cases present diagnostic challenges, and if left unrecognized, the consequences can be life-threatening. We report a rare and atypical case of severe symptomatic hypokalemia as the initial presentation of newly diagnosed Graves’ disease. The condition was caused by thyrotoxic periodic paralysis, a rare but serious complication of thyrotoxicosis, predominantly seen in East Asian populations. This disorder is characterized by episodes of acute, reversible muscle weakness associated with transient hypokalemia, which increases the risk of falls and traumatic injuries. The prompt identification of the etiology in such cases is critical for preventing recurrence and avoiding potentially fatal complications.

## 1. Introduction

**Figure 1:**
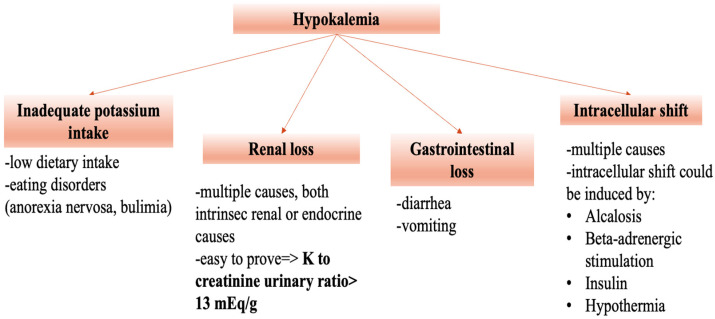
Mechanisms of hypokalemia.

Hypokalemia is one of the most frequently encountered electrolyte disturbances in clinical practice. The mechanisms involved in the development of hypokalemia are a low intake of potassium, renal or digestive losses, and intracellular shift [1] (Figure 1). The etiology is usually identified after medical history review, a physical exam and usual laboratory tests, but in certain cases, multiple tests are necessary for the differential diagnosis.

Most cases of hypokalemia are mild, with obvious, easily treatable causes and with mild or no symptoms. Severe hypokalemia is rarer and may lead to paralysis or dangerous cardiac arrhythmias. The diagnosis of the etiology of hypokalemia is crucial for preventing further episodes and potential life-threatening complications.

Thyrotoxic periodic paralysis is an acquired form of hypokalemic periodic paralysis and represents a rare manifestation of hyperthyroidism. Due to its potential for sudden onset, it can present as the initial manifestation of hyperthyroidism, even in patients with minimal or atypical clinical signs [2]. Given the severity of its clinical presentation and its possible curability, it is imperative for clinicians to recognize this condition as a possible cause of symptomatic hypokalemia.

## 2. Case Report

A 23-year-old Chinese male presented to the Emergency Department of the Clinical Emergency Hospital of Bucharest (Romania) with an inability to move his upper and lower extremities, being incapable of maintaining the standing position, associated with discrete diffuse myalgia. The symptoms appeared two hours before presentation, with progressive worsening. The patient denied any other symptoms, prior similar episodes, or a family history of related conditions. He had been diagnosed with acne one month earlier and was prescribed isotretinoin 10 mg once daily. The patient reported no history of smoking, alcohol use, or psychoactive drugs consumption. He claimed to perform office work, but in the past week he had replaced one of his colleagues and performed significant physical work.

On examination, the patient appeared restless and anxious. He was afebrile, tachycardic (110 bpm), and tachypneic (35 breaths per minute), with a blood pressure of 145/90 mmHg and normal oxygen saturation (98% on room air). The physical examination revealed reduced muscle strength in all four limbs, more pronounced proximally than distally, consistent with a quadriparesis. The remainder of the examination was unremarkable, with no signs of dehydration or edema, no palpable masses or lymphadenopathies, and no bowel or bladder dysfunction. The neurologist confirmed decreased muscle tone and diminished deep tendon reflexes, while cranial nerve function and sensory systems were intact, with no signs of meningeal irritation.

The electrocardiogram (ECG) revealed sinus tachycardia, with incomplete right bundle branch block, a prolonged QTc interval of 516 ms and visible U waves. Initial blood tests in the emergency department showed a potassium level of 2 mEq/L, with no other significant abnormalities. The arterial blood gas analysis demonstrated a mixed metabolic and respiratory alkalosis (pH 7.52, pCO_2_ 29 mmHg, HCO_3_^−^ 30 mEq/L), likely due to hypokalemia and anxiety-induced hyperventilation. At this moment, we reconfirmed that the patient had not experienced emesis or diarrhea, was not taking any other medications, and denied having a personal or family history of disease associated with hypokalemia or paralysis. Due to the patient’s significant restlessness and anxiety, a urinary drug screen was performed, yielding a negative result, thus ruling out psychoactive substances as the cause of hypokalemia. The potassium-to-creatinine ratio in the urine was 11 mEq/g (<13 mEq/g), excluding significant renal potassium loss (Table 1).

Given the fact that the serum potassium level dropped further after the infusion of 500 mL of Ringer lactate (to 1.8 mEq/L), the patient was admitted for treatment. A central venous line was inserted for the administration of potassium chloride at a controlled rate of 10 mEq/h, along with 5 mL of magnesium sulfate 25% solution. After 4 h, the potassium level rose to 2.55 mEq/L, with a significant improvement in muscle strength. The rate of potassium supplementation was then gradually decreased. After 12 h, the K level reached 4 mEq/L, the patient regained full muscle strength and the neurological exam was normal. The ECG showed a normal QTc interval of 390 ms, without U waves. The intravenous infusion of potassium was stopped, and oral potassium salts were continued with close monitoring. After 36 h, the patient developed hyperkalemia (K = 6 mEq/L), but without clinical or ECG changes. Oral supplementation was discontinued and 5 mL of calcium gluconate 10% was administered intravenously. Subsequent potassium levels remained within a normal range.

Given the fact that inadequate potassium intake and digestive and urinary losses were excluded, we concluded that the hypokalemia was likely due to intracellular potassium shifting. Common causes of intracellular shift, such as a primary cause of metabolic alkalosis, beta-adrenergic stimulation or situations associated with an excess of insulin or hypothermia, were ruled out. Therefore, hypokalemic familial periodic paralysis, an autosomal dominant genetic channelopathy, was considered. However, the patient had no history of similar episodes, nor did any family members, and his first episode occurred at 23 years old.

Upon having a closer look, during hospitalization, the patient remained persistently tachycardic at rest (100–110 bpm), with mild hypertension (systolic arterial pressure of 140–150 mmHg), anxiety, and bilateral hand tremor even after potassium levels were corrected. Since hyperthyroidism could be an acquired cause of hypokalemic periodic paralysis, thyroid function tests were performed, which revealed marked hyperthyroidism: TSH 0.015 uIU/L, fT4 60.5 pmol/L (upper normal value 22 pmol/L) and fT3 15.8 pmol/L (upper normal value 6.8 pmol/L), with positive anti-TSH receptor antibody (TRab) determination of 2.12 IU/L (upper normal value 1.75 IU/L) and thyroid peroxidase antibodies (ATPO) of 64 IU/mL (upper normal value 34 IU/mL). A thyroid ultrasound revealed a heterogeneous thyroid echotexture, an absence of nodular lesions and hypervascularity on Doppler imaging, confirming the diagnosis of Graves’ disease. The endocrinologist recommended starting thiamazole 10 mg t.i.d. initially, then reducing it to 10 mg b.i.d. after the first two weeks, with further dosage adjustments based on laboratory results. A propranolol dose of 20 mg b.i.d. and oral supplementation with potassium and magnesium salts were also initiated. Upon discharge, the patient was advised to follow up with the endocrinologist to ensure the achievement of the euthyroid state, crucial for preventing further hypokalemic paralytic episodes. He was also advised to avoid potential triggers, including stress, heavy physical exertion or carbohydrate-rich meals.

**Figure 2:**
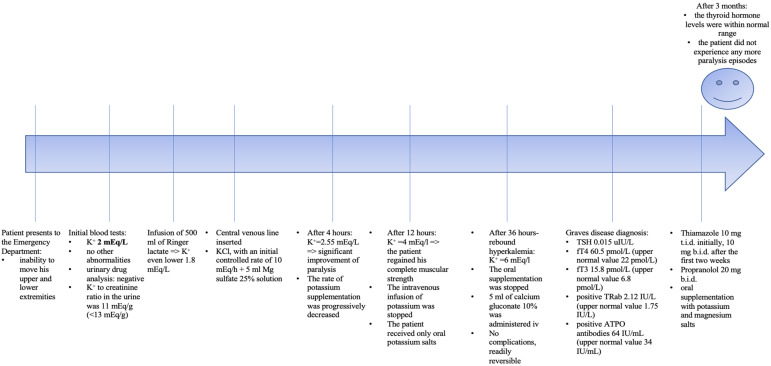
A schematic representation of the timeline of our case.

After three months, the thyroid hormone levels were within a normal range and the patient had not experienced any more paralysis episodes (Figure 2).

## 3. Discussion

Thyrotoxic periodic paralysis (TPP) may be the initial manifestation of Graves’ disease. TPP is a rare yet potentially dangerous complication of thyrotoxicosis, characterized by episodes of acute and reversible muscle weakness due to transient hypokalemia. This condition poses an increased risk of falls, traumatic injuries and, in severe cases, paralysis of the respiratory muscles, leading to acute respiratory failure [2,3]. Factors known to precipitate these attacks include stress; medications such as insulin, diuretics and corticosteroids; intense physical activity; fasting; and carbohydrate-rich meals [3,4]. Our patient reported consuming carbohydrate-rich meals and engaging in unusually intense physical labor before the onset of the attack.

Most cases of hypokalemic periodic paralysis (HPP) are inherited, typically following an autosomal dominant pattern, with mutations identified in genes encoding several ion channels such as calcium voltage-gated channel subunit alpha 1 S (CACNA1S), sodium voltage-gated channel alpha subunit 4 (SCN4A) and potassium voltage-gated channel subfamily E regulatory subunit 3 (KCNE3) [5]. This condition is termed familial hypokalemic periodic paralysis. However, acquired cases of HPP linked to hyperthyroidism have also been reported. TPP is more prevalent in males than in females, despite the higher incidence of thyrotoxicosis in females. It also has a higher incidence in East Asian populations, with a prevalence of 2% in hyperthyroid patients of Asian descent, compared to 0.1–0.2% in the general population [2,6].

Approximately 80% of patients with TPP experience symptoms in the third decade of life. This later age of onset helps to differentiate TPP from familial periodic paralysis, which frequently becomes manifest in the first two decades of life and is considered more often the cause of periodic paralysis in Caucasian individuals and Western countries. However, there have been documented cases of TPP in adolescents [6]. In particular, our patient of Chinese descent presented with his first episode at the age of 23 years old.

In 75% of TPP cases, the hypokalemic crisis is the first clinical manifestation of hyperthyroidism [3]. Because of the condition’s rarity and potential lack of awareness, the diagnosis of TPP is often delayed, with nearly half of patients having experienced multiple episodes before being diagnosed [7]. In our case, this was the patient’s first such episode, with the only suggestive signs of hyperthyroidism being tachycardia, elevated blood pressure, fine hand tremor and anxiety. These symptoms are rather nonspecific and could easily have been attributed to anxiety induced by sudden paralysis.

Virtually any cause of hyperthyroidism could induce TPP, with Graves’ disease being the most prevalent. Other etiologies causes include toxic nodular goiter, solitary toxic nodule, amiodarone-induced thyrotoxicosis, thyrotropin-secreting pituitary adenomas, thyroiditis, and iodine-induced thyrotoxicosis [2,4].

The exact mechanisms underlying hyperthyroidism-induced hypokalemia are not fully understood. Thyroid hormones appear to sensitize the sodium–potassium ATPase pump, leading to a massive intracellular shift of potassium [8,9]. In addition, excess thyroid hormones may enhance the responsiveness of peripheral tissues to adrenergic stimulation, thereby amplifying the hypokalemic effects of catecholamines or insulin [9]. This could explain the role of well-documented triggers, such as carbohydrate-rich meals, stress and physical exertion, which were also involved in our case.

The treatment of TPP involves the management of acute paralytic crisis and definitive long-term care.

During hypokalemic crises, the primary goals are to limit intracellular potassium shifting and replace the potassium deficit [9]. Intravenous potassium chloride is commonly used in clinical practice to quickly raise serum potassium levels and reverse paralysis. However, as the pathophysiology involves the redistribution of potassium rather than a true potassium deficit, aggressive potassium replacement can lead to rebound hyperkalemia [5,9]. Potassium chloride infusion should be administered at a low rate (<10 mmol/h) to avoid rebound hyperkalemia [10]. Despite careful monitoring and adjustments of infusion rates, our patient developed this complication following repletion. Glucose infusions should be avoided, as they elevate insulin levels and exacerbate hypokalemia [3].

Catecholamines’ effect on ion channels can be inhibited by non-selective beta-blockers like propranolol [5]. Beta-blockers inhibit the primary mechanism of these crises, and they should be the first line of treatment in TPP with subsequent paralytic episodes and, in cases of symptomatic acute hypokalemia, when intracellular shift is the primary mechanism.

The definitive treatment for TPP is the achievement of a euthyroid state. In Graves’ disease, this is accomplished with antithyroid medications such as thiamazole. Long-term management should follow the European Thyroid Association Guidelines, which recommend antithyroid drugs as first-line therapy, with definitive treatments such as thyroidectomy or radioactive iodine therapy reserved for patients who do not achieve adequate control with oral medication [9]. Conservative management alone has limited efficacy, with 56% of patients experiencing relapse within seven months of treatment [7,11,12]. In our case, the patient did not experience further paralysis episodes over a three-month follow-up period.

Oral propranolol has also been shown to prevent recurrent paralytic episodes [13]. Therefore, several authors advocate for its use until a euthyroid state is achieved, alongside advice to avoid known precipitating factors such as carbohydrate-rich meals, emotional stress and intense physical exertion [3,5,9].

## 4. Conclusions

In conclusion, thyrotoxic periodic paralysis (TPP) should be considered in patients presenting with hypokalemia and neurological symptoms following physical exertion. Due to similarities in clinical presentation, TPP is frequently misdiagnosed as familial periodic paralysis, which is typically inherited in an autosomal dominant pattern. However, biochemical testing and the presence of thyrotoxic features can aid physicians in differentiating between the two conditions.

## Figures and Tables

**Table 1:** Initial blood tests performed in the emergency department—significant results.

Parameter	Result	Reference Range	Units
Hemoglobin	15.5	11.7–17.4	g/dL
Leukocytes	7000	6000–10,000	/µL
pH	7.52	7.35–7.45	
pO_2_	119	83–108	mmHg
pCO_2_	29	35–48	mmHg
Bicarbonate	30	22–28	mEq/L
Na^+^	142	136–145	mEq/L
K^+^	2	3.4–4.5	mEq/L
Ca^2+^	1.2	1.15–1.27	mmol/L
Urinary K^+^-to-creatinine ratio	11	<13	mEq/g

## Data Availability

Data are contained within the article.
